# Report of Sixty-Nine Cataract Operations

**Published:** 1876-09

**Authors:** A. W. Calhoun

**Affiliations:** Professor of Diseases of the Eye and Ear in the Atlanta Medical College. Atlanta, Ga.


ATLANTA

MEDICAL AND SURGICAL JOURNAL

VOL. XIV.] SEPTEMBER—1876. [No. 6

### Original Communications.

### REPORT OF 69 CATARACT OPERATIONS, MADE, IN THE MAJORITY OF CASES, ACCORDING TO GRAEFE'S LINEAR EXTRACTION.

BY A. W. CALHOUN, M.D.,

PROFESSOR OF DISEASES OF THE EYE AND EAR IN THE ATLANTA MEDICAL COLLEGE.

Ophthalmologists had for many years been zealously seeking some mode of operating, by which the many and grave dangers which had hitherto attended the extraction of cataract, might be greatly diminished, if not entirely overcome. A little more than ten years ago, Von Graefe perfected and gave to the profession what is now known as "Graefe's Linear Extraction," (sometimes called "Graefe's method,") and so nearly has this plan of operating filled the requirements, long and eagerly sought for by oculists, that it remains to the present time the acknowledged method by which the best results are attained, and the smallest number of evils encountered. The operation is by no means perfect in every particular, for doubtless each operator can call to mind that at some special moment in the progress of an operation, he has been made painfully cognizant of the imperfection of a particular step, to correct which, may have cost him much patient but useless toil. But despite the best efforts of each and all,

Graefe's method still offers assurances of success beyond that proposed and thoroughly tested by any other operator.

Hence it is that in most of the reports upon cataract operations from different operators, the linear extraction is the one most usually adopted, and, for the same reason, also, have I been led into its adoption, modifying perhaps certain points here and there to meet sudden necessities that may have arisen in different cases.

It should be mentioned just here, that not all of the 69 operations were made by extraction, but a small portion of them, in children, for instance, with one or two exceptions, come under the head of discision, or laceration. However, the extraction having been made in the large majority of cases, and also, being the operation applicable to the disease in adults, in whom cataract most usually occurs, it is desirable to give it the most prominent place in the report, bringing in the other (the laceration) in its proper turn.

I trust it will not be inappropriate to note what experience has taught me to be of special service in going through the different steps of the operation, for after all, it is in taking care of the little things, that much of the success attending this operation must depend. One who has not accustomed himself to operating with each hand, cannot properly appreciate the advantages had by him who is ambidexter in such operations. The real comfort of knowing, that being able to operate with either the right or left hand, spares you many an uncomfortable position and many an unskilful assistant, is sufficient reason for undergoing much wearisome practice in order to gain such manual dexterity.

I have found that sitting in front of the patient, upon the edge of the bed, and operating upon the left eye with the right hand, and the right eye with the left hand, to be by far the most convenient position both for the operator and the patient. The assistant then stands behind the head and is entirely out of the way. The patient should occupy during the operation, the same bed he is expected to remain in afterwards. This rids the operation of much unnecessary trouble and danger, which often accompanies the removal of the patient from the operating couch, or from one room to another.

It requires no small amount of practice to enable one to make the operation in all its details, exactly as laid down and practiced by Graefe, therefore the slight modification oftentimes mentioned in many of the reports of extraction of cataract.

As being of immense importance to the complete success of the operation, the utmost attention should be given to the formation of the conjunctival flap and the making of the iridectomy. As the knife is brought out above, just behind the corneo-sclerotic junction, a fold of conjunctiva is caught upon its edge and a small flap formed, the value of which cannot well be over-estimated. After the termination of the operation, the early healing of this external cut is very essential to the rapid recovery of the case, and, to a great degree, to the success of the operation. The conjunctival flap, when replaced, overlaps the wound upon the border of the sclera, heals by first intention and keeps the parts in perfect apposition. Not unfrequently, on the day following the operation the flap is found firmly united. Another advantage in the conjunctival flap, is that it prevents the slight but constant outflow of aqueous humour through the wound, which sometimes takes place where no flap has been made, thus interfering with the re-filling of the anterior chamber. Occasionally, also, there is a tendency in the vitreous humour, more particularly when softer than natural, to protrude into the wound and retard the knitting together of the lips of the incision, if not acting as a source of inflammation. The conjunctival flap offers a direct barrier to such a complication, and is therefore an additional assurance of recovery without undue inflammation. As Prof. Knapp very properly remarks: "It enjoys similar advantages to the subcutaneous division of tendons, or the fracture of bones unaccompanied by injury of the skin."

If the extraction be made strictly in accordance with Graefe's method, the conjunctival flap results almost of necessity; otherwise this important part of the operation is lost, and with it goes much that contributes to its ultimate success.

In entering the point of the knife into the anterior chamber, should it be caught in the iris, it is well to withdraw it until it is entirely free or disentangled, before passing it across to the counter-puncture; for so little of the aqueous humour escapes that there is no interference with the movements of the knife. Many authorities insist that the knife should not be withdrawn when once within the anterior chamber, until the termination of the incision, but almost every operator has doubtless noticed that a strict observance of this advice has more than once led to a result which at least delayed, if not directly injured, the successful ending of the case. To continue pushing the knife forward after its point has fastened in the iris, not unfrequently tears it loose from its periphery (iridodialysis) to a considerable extent, thus inducing such hemorrhage into the anterior chamber as to embarrass the further progress of the operation to no small degree. Sometimes, also, there is secondary hemorrhage from the same cause, which gives quite a good deal of trouble, and can become the source of severe inflammation. The withdrawal of the knife's point, to the extent of its disengagement from the iris, is done without the least danger, and is very essential to the satisfactory continuance of this step of the operation.

After the *counter-puncture* has been made, if the iris *then* falls over upon the cutting edge of the knife, the incision should be continued, notwithstanding a piece may be clipped out as the instrument makes its exit at the sclerotic border. This may produce slight hemorrhage, but nothing compared to that which follows the tearing loose of the periphery of the iris.

For somewhat the same reason should the eye be steadily fixed by a careful assistant at the moment the iridectomy is made, for the involuntary, irregular rolling movement of the eye when not fixed or steadied, causes the operator to partly cut and partly tear the iris at its periphery, corresponding to that portion caught up by the forceps for excision, and as a result, more or less copious bleeding follows, obstructing the view. If possible, the blood should be removed from the anterior chamber at once, as clotting takes place in a few minutes, making its removal very difficult and hazardous indeed. This is best done by pressing backwards the sclerotic edge of the wound with a Daviel's spoon, thus allowing the re-accumulated aqueous to flow off and wash the blood out with it.

According to my experience, hemorrhage following the iridectomy, is by far most frequent and most severe in old persons, due perhaps to a want of contractile power in the vessels, and when at all excessive in such individuals, is rather indicative of a tendency to dangerous inflammation.

The legs (edges) of the iridectomy are often left in the scleral wound and become firmly united in it during the healing, acting as a source of iritis, there being a constant traction upon the iris. Hence the necessity of replacing the iris after each operation, by rubbing over the cornea with the closed lid, or by introducing a stilet into the wound and gently loosening from the corners, the cut edges of the iris.

The capsule of the lens should be freely opened, which is best done by passing the cystitome through the capsule in four directions, cutting it in the shape of a square, as it were. Under such circumstances a large portion of it comes away with the lens, but if it is not sufficiently divided, not only is the delivery of the lens made difficult, but the entire partially divided capsule itself, or more or less of it, with portions of the cortical substance of the lens, remains behind and can become the source of secondary cataract. It is an easy matter, by a little unskilfull or careless pressure in the division of the capsule, to dislocate the lens to such a degree that an escape of vitreous humour follows, and sometimes accompanies the attempt to force its exit. Should such a complication take place before the delivery of the lens, the spoon should be at once passed into the eye behind the lens and the latter gently but quickly lifted out. Experience has taught me, however, that the introduction of instruments into the interior of the eye with the view of removing the lens, or any remnant of it, is always a serious matter, and should be avoided when possible to do so.

With regard to the escape of vitreous *after* the removal of the lens, I am convinced that ophthalmologists have allowed themselves to be too easily and too much frightened, and while I would not undervalue the inconvenience of such a complication, I can but still believe that the real danger of it has been much over-estimated. I have in a former publication called attention to this fact, since when, as my experience has extended, I have been more and more satisfied of its truthfulness. It must be confessed that the loss of vitreous *prior* to the removal of the lens is truly a great misfortune, for it *may* defeat the further progress of the operation. Also it must be admitted that in many instances its escape *after* the delivery of the lens is a very great inconvenience, for in cases where a remnant of the cortical substance is left behind, the efforts to dislodge it must usually cease for fear of too great a loss. But that it specially or directly endangers the success of the operation, or is an additional source of inflammation, has not occurred to me in any one or all of my operations. On the contrary, I have often observed the recovery of such cases to be just as prompt and the vision just as good, as of those in whom no such complication existed. At least, there is a shade of doubt thrown around the so-called danger, when we recollect that some of the most successful and noted operators make certain peculiar operations for extraction, by which not only does the vitreous frequently escape without harm, but in which its loss is sometimes even favored, with, at all events, supposed benefit. I would not advise the measures for its prevention to be disregarded by any means, nor would I at all advise any operator to invite the loss of vitreous humour, although I am of the opinion that it oftentimes acts as a direct antiphlogistic under certain circumstances; but I must assert, that I have yet to see one single instance in which injury resulted as a consequence of such loss, *per se.* Hence, when a small amount of vitreous humour follows the lens, I do not anticipate any dangerous result, ceteris paribus, but ridding the wound of as much of it as possible, proceed as usual with the after-treatment, expecting, and in my own cases, without exception, receiving just as perfect success, as to the healing process, as though nothing of the kind had transpired.

Another point of importance is worthy of special attention, viz: the replacement of the iris. After the iridectomy and the removal of the lens, the cut edges of the iris are very often caught in the lips of the corneo-sclerotic wound, and held there during the healing process; thus not only drawing the pupil far up towards the corneo-sclerotic border, but exercising a constant traction upon the iris itself, and acting as a prolific source of iritis, which so often endangers the success of the operation. The replacement is sometimes easily accomplished by rubbing the upper lid with a sort of rotatory movement, over the region of the wound, after the withdrawal of the lid-holder or speculum, and giving a little time for the re-accumulation of a certain amount of aqueous humour. Or, if this should fail, introduce a flexible probe-pointed stilet, bent at the most convenient angle, into the wound, and gently detach the portions of iris from the corners, where they are most usually caught. This is a perfectly safe procedure, and is done without fear of irritating the parts, and rids the operation of a very frequent cause of failure.

### THE DIFFERENT OPERATIONS.

Of the 69 operations, there were 50 extractions made according to Graefe's method, and 1 extraction made by the old flap operation. The remaining 18 were in children or young persons, and discision (or laceration) was practiced upon them.

Of the 51 extractions (including the 1 made by the lower flap operation), the progress of 32 (one is now under treatment and progressing normally, making the number 33) was perfectly normal, nothing whatever interfering with a quick recovery and good vision.* The causes preventing the normal healing of the remaining 18 are as follows:

4 had iritis, in part due to remnants of the lens left behind, resulting in secondary cataract. Upon 3 of these, *iridotomy* was subsequently successfully made, giving them vision equal, respectively, to $\frac{1}{5}$, $\frac{1}{3}$, $\frac{1}{5}$. The same operation was made upon the remaining one, but not sufficiently, and hence without success. I have no doubt, however, but that in this case, a large iridectomy would now give tolerable vision.

4 had plastic iritis, with more or less complete closure of the pupil. Of these, 1 had vision equal to $\frac{1}{200}$, which can be much improved by a subsequent operation. Another can only distinguish large objects, on account of the extension of a large pterygium in front of the pupil. A second operation will also here materially increase the vision. Iridectomy was subsequently performed upon the 3d, giving him vision equal to $\frac{1}{40}$. Unsuccessful *iridotomy* was also made upon the last, or 4th, but no reason exists why a subsequent operation may not restore the vision to some degree.

*Vision of cases given in a following table.

1 received a blow in the eye on the fourth night after the operation, resulting in partial rupture of the corneo-sclerotic wound, slight iritis, etc. No test was made upon the discharge of the patient, but he now has very good vision, being able to use the eye quite well, even attending to his own business (planting).

1 died six hours after the operation, which was performed with ease and perfect success, from supposed apoplexy. (Short history given at end of article—case 1.)

3 were made in cases where the cataracts were secondary and due to irido-cyclitis, and hence strictly regarded, should not be classed amongst the other extractions, but for a better location, they are placed here. In each case, moderate vision was at first obtained, but the pupils becoming again closed by a slow subacute irido-cyclitis, this gain in sight was subsequently lost, and nothing further was attempted.

1 had prolapse of the iris, the result of straining, drawing the pupil far up underneath the upper lid. The prolapse was clipped off and an *iridotomy* made, leaving vision equal to $\frac{2}{3}$.

3 had suppuration of the cornea, totally destroying the vision, which immediately after the operation was tested and found good. They were aged respectively, 62, 67, and 84. In this number is also classed the extraction made by the *old flap method.* (Short history at end of article—case 2.) The cataract in one case was traumatic, which may, in part, account for the tendency to suppuration. There was but little or *no* shrinking of the globe (pthisis bulbi) in either case.

1 had traumatic cataract, with extensive opacity of the cornea, the result of a powder explosion. For awhile after the operation, the patient had vision equal to $\frac{1}{100}$, but the corneal opacity increasing, he is now only able to count fingers immediately before him.

Of the 18 operations by discision or laceration, (mostly in children,) the cure of 15 was without the least disturbance whatever, with more or less increased vision in each, the exact test of which, however, could not be obtained in all, (but an estimate has been made,) on account of their age, in some instances, and their intelligence in others, being insufficient for them to give accurate answers.

Of the remaining 3 that healed abnormally, .

2 had partial suppuration of the cornea, beginning around the puncture from the needle. Of these, 1 can no doubt be yet restored to tolerable vision by making an iridectomy behind the clear portion of cornea, and removing the remnant of lens. The other was in a person addicted to drink, who, by continuing his habit, has kept the eye in a constant state of irritation, rendering the propriety of a second operation very doubtful; their ages are 18 and 27.

1 had suppuration of the entire cornea, beginning as in the other cases, around the needle puncture, resulting in complete sloughing of the cornea up to the sclerotic border. There was, however, no shrinking of the ball, nor was there any, as before remarked, in any of the entire number of operations (69). The age of this last case was 31. (Short history at end of article—case 3.)

These 3 operations just mentioned as progressing abnormally, were in the oldest of the entire number of persons upon whom laceration was performed, and in each case the needle was entered *through the cornea.* My experience, though limited, teaches me two things in regard to this operation, viz: that it is safest in children before reaching puberty, the danger increasing year by year from this time on. Also, that it is frequently unsafe to enter the needle *through the cornea,* but *scarcely ever, through the sclerotic*, passing it between the lens and the iris. The reasons for all this are obvious. I have frequently made the operation through the sclerotic and have never had the slightest trouble to follow, and have invariably found it easier of performance, since in this position greater play can be given the needle. In my opinion, it is particularly applicable when a repetition of the operation is required, as is usually the case in soft cataract, where the lens has already been partially broken in pieces by the first operation.

The ages of the 18 (leaving out the 3 before mentioned, aged 18, 27, and 31, and still another aged 20) ranged from 5 to 13 years, and the vision of each (again excluding the same 3) was more or less increased, though in a few only slightly, while in others very prominently so, and indeed in some, the restoration to normal vision was almost complete. Children with cataract should be operated upon very early in life, and the fact that they are not, oftentimes renders the operation very nearly useless, because of the atrophy of the optic nerve which necessarily follows its continuous non-use, brought about by a cataractous lens preventing the entrance of light into the interior of the eye, and the proper exercise of the nerve. A single glance with the ophthalmoscope at the optic nerves of children who have had cataract, *especially if congenital*, will in many instances easily demonstrate the reason why an operation, perfectly successful in itself, is so often crowned by such negative results. I have, in several instances been able to benefit the condition of the nerve and thereby increase the vision, by the free and persistent use of strychnine, with, in some cases, the systematic use of electricity. This treatment is, at any rate, worthy of trial in every instance where such a condition of things exist. One should be particularly guarded about giving a favorable prognosis in cases of *congenital* cataract, if such is based upon the fact that the little patient has good perception of light, can distinguish colors, etc.; for not unfrequently after the complete absorption of the lens, the vision is but little if any better than before the operation.

In summing up the results, I have for convenience sake, placed the cases under four different headings,* viz:

1st. *Complete success*, with vision ranging from $\frac{1}{10}$ to $\frac{1}{1}$, with which patients can, with proper glasses, attend to most of the ordinary duties of life, and in the most successful cases, read and write.

2d. *Half success*, with vision ranging from $\frac{1}{200}$ to $\frac{1}{10}$, with which, in the most unfavorable cases, the patients can go about alone, and in the favorable, are able to do ordinary work, and read very large type.

3d. *Curable blindness*, in which a subsequent operation will in all probability restore more or less vision, inflammation having marred the good effects of the first.

4th. *Incurable blindness*, in which nothing more can be done for the relief of the individual, the operation having proved to be a failure.

Taking first the 51 operations by extraction (including the 1 by the flap method), and afterwards those by laceration, we have, (of the 51):

*According to Knapp's arrangement.

| | | | | | | |
|---|---|---|---|---|---|---|
| Complete success | in | 37* | cases, | that is, | 72.55 | per cent. |
| Half | " " | 5 | " | " | 9.8 | " |
| Curable blindness | " | 2 | " | " | 3.921 | " |
| Incurable " | " | 3 | " | " | 5.882 | " |
| †Complicated | " | 3 | " | " | 5.882 | " |
| Death from apoplexy 6 hours after operat'n | " | 1 | " | " | 1.96 | " |

It should be borne in mind that several of those cases in the above table, under the head of "complete success," had progressed abnormally after the extraction, and required a second operation to bring them into this class and condition of completeness. The "complicated" cases being cataract resulting from irido-cyclitis, *should not* be brought into the *general estimate of results*, but I add them here in order to make the list complete. Some good was accomplished in each instance by the operation, but it was again lost by a revival of the irido-cyclitis in its acute form. The "death" in the one case, is added here for want of a better place to locate it.

Of the 18 operations upon young persons and children (made by discision or laceration), we have, classing them in the same way as the extractions:

| | | | | | | |
|---|---|---|---|---|---|---|
| Complete success | in | 9 | cases, | that is, | 50 | per cent. |
| Half | " " | 6 | " | " | $33\frac{1}{3}$ | " |
| Curable blindness | " | 1 | " | " | $.05\frac{5}{9}$ | " |
| Incurable " | " | 2 | " | " | $11\frac{1}{9}$ | " |

Excluding the last 3 cases, all had more or less increased vision, but the 9 (under "complete success") had a remarkable increase of it, enabling them, in a few instances, to see sufficiently clearly to read or write, and the same good result would have been accomplished in the others under this heading, had they been mentally able, or rather, had they have had proper practice in such exercises. Those who have had much experience in operating upon cataract in children, will at once appreciate the success gained in the above entire number of operations.

An iridectomy and the removal of the remaining portion of

---

*One is now under treatment, but has progressed far enough and so favorably as to make it an assured success.

†Cataract the result of irido-cyclitis.

lens will soon be made upon the case of "curable blindness," with every prospect of success.

In regard to the effect of age upon the progress and success of the operations, (extraction and laceration,) I am inclined to the opinion that, as a rule, other things being equal, the old get well just as satisfactorily as those younger, and the only difference in the result, is that attributable to the failure of vision naturally connected with the increase in number of years. The following table (including children and adults) will show the influence that age may have had in deciding the good or bad termination (that is, the vision) of the case:

| Age. | No. of cases. | Vision. | | | Remarks. |
|---|---|---|---|---|---|
| | | Good. | Medium. | Bad. | |
| Betw'n 1 and 10 yrs | 8 | 5 | 3 | | All 15 were by laceration or discision. The vision of the "bad" one can be increased by a 2d operation. |
| " 10 " 20 " | 7 | 2 | 4 | 1 | |
| " 20 " 30 " | 4 | 3 | | 1 | Vision can also be improved by a 2d operation in 1 "bad." |
| " 30 " 40 " | 4 | 3 | | 1 | |
| " 40 " 50 " | 7* | 3 | 1 | (3)† | The 3 "bad" are the complic't'd cases before mentioned — vision benefited but again lost. The 1 "medium" is the traumatic cataract by explosion. Vision diminished subsequently. |
| " 50 " 60 " | 9 | 9 | | | |
| " 60 " 70 " | 10 | 7 | 1 | 2 | |
| " 70 " 80 " | 18 | 14 | 2 | 2 | One of the "bad" can be benefited by a 2d operation; one of the "good" was she that died from apoplexy. |
| " 80 " 90 " | 2 | 1 | | 1 | |
| | 69 | 47 | 11 | 11-3=8 | |

* Including the one now under treatment.

† Complicated cases which should not be placed in the general estimate.

It will be seen that the number of cases in this table under "good vision," exceed those in the other tables under "complete success" by one. This is due to the fact that under the head of "good vision" I have placed the case which died in a few hours after the operation, evidently from apoplexy. The operation was made without the least trouble, and everything was progressing quite smoothly up to the time of death. For this reason it is classed under this heading. The very worst of those under "medium vision" can go about unattended, and the most of them are able to attend to the usual every day duties of life.

Under "bad vision" are the 3 cases of cataract resulting from irido-cyclitis, each of which was, to some degree, improved by the extraction, but the amount of sight thus gained was subsequently lost by an acute attack of the original disease. Deducting these 3 from the general estimate of results, there still remain 8 under bad vision, 3 of which *are* "*curable,*" by a large iridectomy being made, forming an artificial pupil. There are then only 5 absolute failures, or 7.246 per cent. of the whole number, and 3 *partial* failures, (curable blindness,) the latter being still amenable to treatment. In short, a success of 92.754 per cent. of the whole number.

The influence of age upon the success of the operations can be seen at a glance at the table, the *greatest percentage* being between 50 and 60.

The duration of the treatment in the different cases was quite variable, as can be seen from the following table.

They were dismissed from treatment as follows:*

| | | | |
|---|---|---|---|
| 2 | after | 6 | days. |
| 5 | " | 7 | " |
| 4 | " | 8 | " |
| 2 | " | 9 | " |
| 2 | " | 10 | " |
| 2 | " | 15 | " |
| 6 | " | 16 | " |
| 2 | " | 17 | " |
| 4 | " | 18 | " |
| 2 | " | 19 | " |
| 5 | " | 20 | " |
| 9 | " | 21 | " |
| 2 | " | 23 | " |
| 4 | " | 24 | " |

* Exclusive of the 1 now under treatment.

| | | | |
|---|---|---|---|
| 1 | after | 26 | days. |
| 3 | " | 27 | " |
| 1 | " | 29 | " |
| 6 | " | 30 | " |
| 1 | " | 35 | " |
| 1 | " | 43 | " |
| 3 | " | 45 | " |
| 67 | " | 1347, | " |

making the *average* time under treatment, that is, before their dismissal, a little more than 20 days for the different cases.

The case of sudden death is left out of this table, inasmuch as no change of importance is produced thereby, in making up the general average. The cases of discision are also here included, and it must be mentioned that upon most of them a second, and upon a few a third, operation was necessary, but the length of time that they were kept under treatment, after each, was about the same, and corresponds to the smallest number of days in the table.

Below is a table showing the amount of vision had by the different cases after the operation (upon their dismissal), tested with the aid of double convex (cataract) glasses. They are arranged in groups, several having the same amount of vision:

| | | | | | |
|---|---|---|---|---|---|
| 2 | had | vision | equal | to | 1. |
| 5 | " | " | " | " | $\frac{2}{3}$. |
| 5 | " | " | " | " | $\frac{1}{2}$. |
| 4 | " | " | " | " | $\frac{2}{5}$. |
| 4 | " | " | " | " | $\frac{1}{3}$. |
| 3 | " | " | " | " | $\frac{1}{4}$. |
| 5 | " | " | " | " | $\frac{1}{5}$. |
| 6 | " | " | " | " | $\frac{1}{7}$. |
| 7 | " | " | " | " | $\frac{1}{8}$. |
| 4 | " | " | " | " | $\frac{1}{10}$. |
| 2 | " | " | " | " | $\frac{1}{12}$. |
| 1 | " | " | " | " | $\frac{1}{15}$. |
| 1 | " | " | " | " | $\frac{1}{20}$. |
| 2 | " | " | " | " | $\frac{1}{40}$. |
| 1 | " | " | " | " | $\frac{1}{50}$. |
| 1 | " | " | " | " | $\frac{1}{75}$. |
| 2 | " | " | " | " | $\frac{1}{100}$. |
| 1 | " | " | " | " | $\frac{1}{200}$. |
| 1 | not yet tested. | | | | |

The remaining 12 cases are made up of the 3 "complicated" (viz: cataract resulting from irido-cyclitis, which again appeared acutely after the operations, and destroyed what little vision was gained thereby—not to be classed in the *results*), the 1 in which death followed several hours after the operation, which in itself was perfectly satisfactory; the 3 (partial successes, or curable blindness) which still require secondary operations to give them good vision, and the 5 resulting in complete failure, making, with those enumerated in the above table, the entire number (69) of cases.

In operating upon these cases, no regard whatever has been paid to the season of the year in which they presented themselves, so that indeed every season and every month have been thoroughly tested, so to speak, and as yet I have seen no reason for prefering one season or one month to another, with the view of affording a more favorable progress or a better termination of the cases. The heat of summer makes the necessary confinement of about a week to the bed and room quite uncomfortable to the patient, but it does not exert the least influence upon the ultimate result of the case. In short, so far as the success of the operation itself is concerned, no particular time or month seem to possess any special advantages over another.

In order that, at a glance, a succinct history of each case may be had, I have appended below a table, showing in each one, *the age, length of time blindness has existed, character of the cataract, method of operating, progress of the case after operation, length of time under treatment, acuity of vision, and the glasses necessary for near and distant seeing:*

| No. | Age. | Blind for | Eye, R or L | Character of Cataract. | Method of Operating. | Progress of Case | Under Treatment. | Vision | Glass for Dist. | Glass for Reading. | REMARKS. |
|---|---|---|---|---|---|---|---|---|---|---|---|
| 1 | 82 | 6 yrs. | L | Mature Senile, or Nuclear | Upper Flap Ext'n. | Normal. | 16 days. | $\frac{1}{8}$ | $+\frac{1}{3\frac{1}{2}}$ | | |
| 2 | 70 | 4 " | L | " " | " " " | " | 24 " | $\frac{1}{4}$ | $+\frac{1}{4}$ | | |
| 3 | 60 | 3 " | L | " " | " " " | " | 18 " | $\frac{1}{2}$ | $+\frac{1}{4}$ | $+\frac{1}{2\frac{1}{2}}$ | |
| 4 | 60 | 2 " | R | " " | " " " | Abnormal. | 30 " | $\frac{1}{40}$ | $+\frac{1}{3}$ | ...... | Had iritis, with partial closure of pupil. |
| 5 | 5 | 5 " | R | Congenital. | Discision. | Normal. | 7 " | $\frac{1}{10}$ | | | |
| 6 | 26 | 26 " | R | Lamellar (cong'tl) | Upper Flap Ext'n. | " | 20 " | $\frac{2}{5}$ | $+\frac{1}{3\frac{3}{4}}$ | $+\frac{1}{2\frac{1}{2}}$ | |
| 7 | 70 | 3 " | L | Mature Senile, or Nuclear | " " " | " | 23 " | $\frac{1}{5}$ | $+\frac{1}{4}$ | $+\frac{1}{2\frac{1}{4}}$ | |
| 8 | 50 | $2\frac{1}{2}$ " | L | " " | Graefe's Ext'n. | " | 19 " | $\frac{1}{8}$ | $+\frac{1}{3\frac{1}{2}}$ | | |

| | | | | | | | | | | | |
|---|---|---|---|---|---|---|---|---|---|---|---|
| 9 | 10 | 10 yrs. | R | Cortical (cong'tl). | Discision. | Normal. | 15 days. | $\frac{1}{12}$ | | | |
| 10 | 40 | $4\frac{1}{2}$ " | R | Mature Senile, or Nuclear | Graefe's Ext'n. | Abnormal. | 29 " | $\frac{2}{3}$ | $+\frac{1}{4\frac{1}{2}}$ | $+\frac{1}{2\frac{3}{4}}$ | Iritis with pupil occlusion. Successful iridotomy. |
| 11 | 72 | $1\frac{1}{2}$ " | R | " " | " " | Died 6 h'rs after operation. | | | | | |
| 12 | 43 | 1 " | R | Secondary. | " " | Abnormal. | 27 " | .... | .... | ...... | Cataract resulting from irido-cyclitis. Some vision after operation, but lost by a return of the original trouble. |
| 13 | 5 | 5 " | R | Cortical (cong'tl.) | Discision. | Normal. | 7 " | $\frac{1}{40}$ | | | |
| 14 | 10 | 10 " | L | " " | " | " | 15 " | $\frac{1}{10}$ | | | |
| 15 | 50 | 4 " | R | Mature Senile, or Nuclear | Graefe's Ext'n. | " | 18 " | $\frac{2}{3}$ | $+\frac{1}{4\frac{1}{2}}$ | $+\frac{1}{2\frac{3}{4}}$ | |
| 16 | 76 | 3 " | R | " " | " " | Abnormal. | 30 " | .... | .... | ...... | Had sub-acute irido-cyclitis, with closure of pupil. |
| 17 | 13 | 13 " | R | Cortical (cong'tl) | Discision. | Normal. | 8 " | $\frac{1}{12}$ | | | |
| 18 | 70 | 2 " | R | Mature Senile, or Nuclear. | Graefe's Ext'n. | " | 18 " | $\frac{1}{8}$ | $+\frac{1}{4}$ | $+\frac{1}{2\frac{1}{2}}$ | |
| 19 | 9 | 9 " | R | Cortical (cong'tl) | Discision. | " | 8 " | $\frac{1}{8}$ | | | |

Vol. XIV.—No. 6.—22.

| No. | Age. | Blind for | Eye, R or L | Character of Cataract. | Method of Operating. | Progress of Case | Under Treatment. | Vision | Glass for | |
|---|---|---|---|---|---|---|---|---|---|---|
| | | | | | | | | | Dist. | Reading. | REMARKS. |
| 20 | 55 | $1\frac{1}{2}$ yrs. | R | Mature Senile, or Nuclear. | Graefe's Ext'n. | Normal. | 21 " | $\frac{1}{1}$ | $+\frac{1}{4}$ | $+\frac{1}{2\frac{1}{2}}$ | Pupil abnormally enlarged before and after operation. |
| 21 | 48 | 2 " | L | Secondary. | " " | Abnormal. | 43 " | .... | .... | ...... | Cataract resulting from irido-cyclitis. Some vision after operation, but lost by return of original disease. |
| 22 | 27 | 7 " | R | Lamellar (cong'tl) | Discision. | " | 35 " | .... | .... | ...... | Partial suppuration of cornea, beginning in needle puncture. |
| 23 | 13 | 13 " | L | Cortical (cong'tl) | " | Normal. | 7 " | $\frac{1}{10}$ | | | |
| 24 | 50 | 2 " | R | Mature Senile, or Nuclear. | Graefe's Ext'n. | " | 21 " | $\frac{1}{3}$ | $+\frac{1}{3\frac{1}{2}}$ | | |
| 25 | 27 | 7 " | L | Lamellar (cong'tl) | " " | " | 20 " | $\frac{1}{3}$ | $+\frac{1}{4}$ | $+\frac{1}{2\frac{1}{2}}$ | |
| 26 | 48 | 2 " | R | Secondary. | " " | Abnormal. | 27 " | .... | .... | ...... | Cataract from irido-cyclitis. Good effect of operation lost subsequently. |
| 27 | 9 | 9 " | L | Cortical (cong'tl) | Discision. | Normal. | 8 " | $\frac{1}{8}$ | | | |
| 28 | 13 | 10 " | L | " " | " | " | 7 " | $\frac{1}{7}$ | | | |

| | | | | | | | | | | | |
|---|---|---|---|---|---|---|---|---|---|---|---|
| 29 | 71½ | 3½ yrs. | R | Mature Senile, or Nuclear | Graefe's Ext'n. | Normal. | 16 " | ⅛ | + ¼ | + 1/2½ | |
| 30 | 58 | 2½ " | R | " " | " " | Abnormal. | 30 " | ⅔ | + ¼ | + 1/2½ | Through straining, a rupture of the wound and large prolapse of the iris followed. Excision of prolapsus and successful iridotomy. |
| 31 | 71 | 3 " | L | " " | " " | Normal. | 19 " | ¼ | + ¼ | + 1/2½ | |
| 32 | 66 | 2 " | L | " " | " " | Abnormal. | 45 " | 1/7 | + 1/3¾ | + 1/2¼ | Had iritis with exudative pupillary membrane. Successful iridotomy. |
| 33 | 15 | 4½mo's | R | Traumatic. | Discision. | Normal. | 10 " | 1/1 | + 1/3¾ | + 1/2¼ | |
| 34 | 62 | 10 yrs. | L | Mature Senile, or Nuclear. | Flap Ext'n (lower) | Abnormal. | 30 " | .... | .... | ...... | Suppuration of cornea followed. |
| 35 | 8 | 8 " | R | Lamellar. | Discision. | Normal. | 7 " | 1/7 | + 1/3 | | |
| 36 | 8 | 8 " | L | " | " | " | 8 " | 1/7 | + 1/3¼ | | |

| No. | Age. | Blind for | Eye, R or L | Character of Cataract. | Method of Operating. | Progress of Case | Under Treatment. | Vision | Glass for Dist. | Glass for Reading. | REMARKS. |
|---|---|---|---|---|---|---|---|---|---|---|---|
| 37 | 18 | 4 yrs. | L | Cortical. | Discision. | Abnormal. | 18 days. | .... | .... | ...... | Partial suppuration of cornea followed. Will require an iridectomy. |
| 38 | 43 | 8 " | L | Mature Senile, or Nuclear | Graefe's Ext'n. | " | 45 " | $\frac{1}{3}$ | $+\frac{1}{4}$ | $+\frac{1}{2\frac{1}{2}}$ | Had iritis and closure of pupil. Successful iridectomy. |
| 39 | 71 | 1 " | R | " " | " " | Normal. | 16 " | $\frac{1}{10}$ | $+\frac{1}{4}$ | $+\frac{1}{2\frac{1}{2}}$ | |
| 40 | 5 | 5 " | L | Congenital. | Discision. | " | 6 " | $\frac{1}{75}$ | | | |
| 41 | 9 | 5 " | L | Cortical. | " | " | 10 " | $\frac{1}{50}$ | $+\frac{1}{5}$ | $+\frac{1}{2\frac{3}{4}}$ | Has scrofulous corneal opacities |
| 42 | 73 | 7 " | R | Mature Senile, or Nuclear | Graefe's Ext'n. | " | 24 " | $\frac{1}{5}$ | $+\frac{1}{3\frac{3}{4}}$ | $+\frac{1}{2\frac{1}{2}}$ | |
| 43 | 73 | 1 " | L | " " | " " | Abnormal. | 26 " | $\frac{1}{15}$ | $+\frac{1}{4}$ | $+\frac{1}{2\frac{1}{4}}$ | Had iritis. |

| | | | | | | | | | | | |
|---|---|---|---|---|---|---|---|---|---|---|---|
| 44 | 70 | 2 yrs. | L | " " | " " | Normal. | 17 days. | $\frac{2}{3}$ | $+\frac{1}{3\frac{3}{4}}$ | $+\frac{1}{2\frac{1}{2}}$ | |
| 45 | 70 | 8 " | R | " " | " " | Abnormal. | 30 " | $\frac{1}{100}$ | .... | ...... | Had iritis, complicated by pterygium partially covering pupil. |
| 46 | 84 | 3 " | L | " " | " " | " | 16 " | .... | .... | ...... | Suppuration of cornea followed. |
| 47 | 52 | 4 " | L | " " | " " | " | 23 " | $\frac{1}{20}$ | $+\frac{1}{4}$ | $+\frac{1}{2\frac{1}{2}}$ | Received blow upon ball before wound healed. |
| 48 | 52 | 4 " | R | " " | " " | Normal. | 9 " | $\frac{1}{3}$ | $+\frac{1}{3\frac{3}{4}}$ | | |
| 49 | 73 | 6 " | R | " " | " " | " | 21 " | $\frac{1}{2}$ | $+\frac{1}{4}$ | $+\frac{1}{2\frac{1}{2}}$ | |
| 50 | 31 | 26 " | R | Chalky. | Discision. | Abnormal. | 17 " | .... | .... | ...... | Partial suppuration of cornea followed. |
| 51 | 31 | 26 " | L | " | Graefe's Ext'n. | Normal. | 30 " | $\frac{1}{5}$ | $+\frac{1}{4}$ | $+\frac{1}{2\frac{1}{2}}$ | |
| 52 | 76 | 3 " | R | Mature Senile, or Nuclear | " " | Abnormal. | 45 " | .... | .... | ...... | Iritis, with closure of pupil. Iridectomy necessary. |
| 53 | 71 | 3 " | L | " " | " " | Normal. | 20 " | $\frac{2}{3}$ | $+\frac{1}{4}$ | $+\frac{1}{2\frac{1}{2}}$ | |

| No. | Age. | Blind for | Eye, R or L | Character of Cataract. | Method of Operating. | Progress of Case | Under Treatment. | Vision | Glass for Dist. | Glass for Reading. | REMARKS. |
|---|---|---|---|---|---|---|---|---|---|---|---|
| 54 | 60 | 2 yrs. | R | Mature Senile, or Nuclear. | Graefe's Ext'n. | Normal. | 21 days. | $\frac{1}{2}$ | $+\frac{1}{4}$ | | |
| 55 | 60 | 1 " | L | " " | " " | " | 16 " | $\frac{2}{5}$ | $+\frac{1}{4}$ | | |
| 56 | 31 | 5 " | R | Traumatic. | " " | " | 24 " | $\frac{1}{5}$ | $+\frac{1}{3\frac{3}{4}}$ | $+\frac{1}{2\frac{1}{2}}$ | A 2d iridectomy had to be made, to increase the size of the pupil. |
| 57 | 67 | 14 " | R | " | " " | Abnormal. | 21 " | .... | .... | ...... | Suppuration of cornea. |
| 58 | 39 | 4 " | R | Mature Senile, or Nuclear | " " | Normal. | 9 " | $\frac{1}{2}$ | $+\frac{1}{4}$ | $+\frac{1}{2\frac{1}{2}}$ | |
| 59 | 48 | 10 " | R | Traumatic. | Flap Ext'n (lower) | " | 21 " | $\frac{1}{200}$ | .... | ...... | Vision partially lost by an increase of an old corneal opacity, after operation, |
| 60 | 66 | 5 " | R | Mature Senile, or Nuclear. | Graefe's Ext'n. | Abnormal. | 21 " | $\frac{1}{100}$ | .... | ...... | Had iritis. |
| 61 | 66 | 6 mos | L | " " | " " | Normal. | 16 " | $\frac{1}{4}$ | $+\frac{1}{4}$ | | |

| | | | | | | | | | | |
|---|---|---|---|---|---|---|---|---|---|---|
| 62 | 758 mo's. | L | Mature Senile, or Nuclear (black) | Graefe's Ext'n. | Normal. | 20 days. | $\frac{2}{5}$ | $+\frac{1}{4}$ | $+\frac{1}{2\frac{1}{2}}$ | |
| 63 | 61 2 yrs. | L | " " | " " | " | 20 " | $\frac{1}{7}$ | $+\frac{1}{3\frac{3}{4}}$ | $+\frac{1}{2\frac{1}{4}}$ | |
| 64 | 2020 " | L | Lamellar. | Discision. | " | 6 " | $\frac{1}{2}$ | $+\frac{1}{3\frac{1}{2}}$ | | |
| 65 | 514 mo's. | L | Mature Senile, or Nuclear. | Graefe'e Ext'n. | " | 21 " | $\frac{1}{5}$ | $+\frac{1}{4}$ | $+\frac{1}{2\frac{1}{2}}$ | |
| 66 | 70 2 yrs. | L | " " | " " | " | 24 " | $\frac{2}{5}$ | $+\frac{1}{4}$ | $+\frac{1}{2\frac{1}{2}}$ | |
| 67 | 554 mo's. | L | " " | " " | " | 27 " | $\frac{1}{7}$ | $+\frac{1}{4}$ | | |
| 68 | 72 2 yrs. | L | " " | " " | " | 21 " | $\frac{1}{8}$ | $+\frac{1}{4}$ | | |
| 69 | 456 mo's. | R | Lens partially cataractous and dislocated into anterior chamber. | Graefe's Ext'n, (lower cut.) | " | ........ | .... | .... | ...... | Vision not yet tested. |

The vision in a few of the cases has been only *estimated*, and not actually tested with test types, etc., due to the several causes already mentioned, but the estimate has been made at carefully and correctly as possible, and then reduced so as to equal the amount of sight given in figures in the table—under vision—and corresponding to the results of such tests as are usually made after cataract operations.

Case 1.—Mrs. M. æt. 72 years, in good health for one of her age, had mature senile or nuclear cataract in each eye, being thus completely blinded for several previous years. A few days prior to the operation, some slight preparatory treatment was practiced, such as attention to the bowels, diet, etc. No anæsthetic, nor opiate of any kind nor any stimulant whatever was given, and the operation—Graefe's linear extraction—was made without the slightest difficulty, the patient expressing herself as having suffered very little pain indeed. After the removal of the lens and the cleansing of the eye, the usual test of the vision, by means of the fingers, was made and found quite satisfactory, she being able to count and distinguish the fingers one from the other with unusual promptness. Upon placing a strong convex glass before the eye, she recognized several of her friends standing near her, and became so extremely excited as a result of it, that it required some little time to quiet her.

After dressing and bandaging the eye, she became nauseated and vomited freely several times. About three hours after the operation she complained of pain in the head, and an hour later became comatose, with that peculiar stertorous breathing so indicative of pressure upon the brain, and, after two hours more, (about 6 hours after the operation,) she died. The cause of death was clearly a rupture of a cerebral blood-vessel, but whether this took place as a result of the excitement following the operation and was the cause of the nausea and vomiting, or whether this latter, with its accompanying straining, etc., produced the rupture of the vessel, is difficult to decide.

Case 2.—Mr. F., æt. 62 years, and in good health, was thrown from a carriage when 20 years old (42 years ago), injuring both eyes by the fall. Slight internal strabismus (double) began a short time afterwards, which gradually increased year by year, until the balls were drawn inwards so far, that the corneæ were entirely hidden behind the internal canthi, and could only be seen by grasping a fold of conjunctiva near the external canthus, and forcibly rolling the eye outwards. Indeed, almost the posterior surface of the balls presented themselves to view. On account of paralysis of the external recti muscles, he had no power whatever to turn the eyes outwards, and was consequently, for all practical purposes, blind.

The internal recti were divided at the same time, and the *advancement* operation was made upon the *external* recti muscles. These operations succeeded in correcting the double squint, but it was found that cataract existed in each eye, mature in the left and immature in the right; and also that there were corneal opacities (perhaps the result of ulceration) partially covering each pupil. By dilating the right pupil, he saw sufficiently well to go about and attend to all ordinary business; in fact, his vision was better than it had been for a great number of years, even though the lens of this eye was partially cataractous.

After the lapse of several months, during which time the eyes had recovered fully from the strabismus operations, the lens of the left eye was extracted by means of the lower flap operation, combining with it an iridectomy. The usual test (with fingers) of vision was made, which was found to be good, notwithstanding this eye had not been used for a long series of years.

On the 3d day after the operation, suppuration of the cornea began, and resulted in the sloughing of a large portion o it. No collapse of the ball (phthisis bulbi) followed, the form, etc., remaining natural.

The upper portion of the cornea remains clear, and perhaps later, an iridectomy will result in the restoration of some vision.

This short history of the case is given, not on account of anything unusual in the cataract, but for the peculiarity of the extent of the strabismus (about equal in both eyes), and the operations for its correction.

Case 3.—Mr. W., æt. 31 years; saw well, as a child, up to his 5th or 6th year, when cataract rapidly formed in both eyes, thus causing complete blindness for about 26 years. He recollects some few things that he had seen in his boyhood, but his memory as to their appearance is vague and uncertain. To all appearances, the lenses were quite soft, and under this belief, the needle operation (discision) was made upon the right eye through the cornea. The needle, however, entered the lens substance with difficulty, due to its unexpected hardness, and consequently only a very small portion of the cortical substance was broken up. Severe inflammation followed around the needle puncture, gradually extending until the entire cornea became implicated, suppurated, and sloughed off.

After the subsidence of the inflammation in this eye, Graefe's linear extraction was made upon the left, removing the lens with perfect ease. The recovery was rapid, and at the end of the second week after this last operation, he was dismissed, being able to count fingers without hesitancy at a distance of 30 feet, with the aid of a double convex glass No. 4 ($+\frac{1}{4}$). His vision has gradually improved, and when recently heard from, he was walking about over his farm, attending to such business as he felt mentally capable of.

It is contrary to expectation—at any rate rather remarkable—that the retina should have resumed so promptly its natural functions, after having been in almost total dis-use for twenty-five or six years. In this instance, it is due, doubtless, to the fact that during the first few years of his life the nerve and retina had undergone the usual normal exercise, and was thus so far strengthened as to be enabled to withstand the effects of the long imprisonment in darkness that followed. There is but little doubt that the judicious exercise of the retina, etc., with properly adjusted glasses, will ultimately result in his restoration to fully $\frac{1}{3}$ or $\frac{1}{2}$ of his normal vision.

